# Genome-wide analysis of acid tolerance genes of *Enterococcus faecalis* with RNA-seq and Tn-seq

**DOI:** 10.1186/s12864-024-10162-z

**Published:** 2024-03-08

**Authors:** Zhanyi Chen, Chenguang Niu, Lifan Wei, Zhengwei Huang, Shujun Ran

**Affiliations:** 1 Department of Endodontics and Operative Dentistry, Ninth People’s Hospital, College of Stomatology, Shanghai Jiao Tong University School of Medicine, Shanghai, China; 2 National Clinical Research Center for Oral Diseases, Shanghai, China; 3 Shanghai Key Laboratory of Stomatology & Shanghai Research Institute of Stomatology, Shanghai, China; 4 Nucleic acid drug Research and Development Institute, CSPC, Shanghai, China

**Keywords:** *Enterococcus faecalis*, Acid tolerance, Acid stress, RNA-seq, TIS

## Abstract

*Enterococcus faecalis*, a formidable nosocomial and community-acquired opportunistic pathogen, can persist a wide range of extreme environments, including low pH and nutrient deficiency. Clarifying the survival mechanism of *E. faecalis* in low-pH conditions is the key to combating the infectious diseases caused by *E. faecalis.* In this study, we combined transcriptome profiling (RNA-seq) and transposon insertion sequencing (TIS) to comprehensively understand the genes that confer these features on *E. faecalis*. The metadata showed that genes whose products are involved in cation transportation and amino acid biosynthesis were predominantly differentially expressed under acid conditions. The products of genes such as *opp1C* and *copY* reduced the hydrion concentration in the cell, whereas those of *gldA2*, *gnd2*, *ubiD*, and *ubiD2* mainly participated in amino metabolism, increasing matters to neutralize excess acid. These, together with the *folE* and *hexB* genes, which are involved in mismatch repair, form a network of *E. faecalis* genes necessary for its survival under acid conditions.

## Importance

As a serious nosocomial pathogen, *Enterococcus faecalis* was considered responsible for large numbers of infections. Its ability to survive under stress conditions, such as acid condition and nutrient deficiency was indispensable for its growth and infection. Therefore, understanding how *E. faecalis* survives acid stress is necessary for the prevention and treatment of related diseases. RNA-seq and TIS provide us a way to analyze the changes in gene expression under such conditions.

## Introduction

*Enterococci*, which occur widely in the human and animal gastrointestinal tracts, have been previously linked to infections of the skin and soft tissues, especially endocarditis and urinary tract infections [1]. *Enterococcus faecium* and *Enterococcus faecalis* are the species of enterococci most strongly associated with disease and are responsible for 97% of infective endocarditis, predominantly affecting the elderly and patients with comorbidities [2]. As well as the infections mentioned above, *E. faecalis* plays a significant role in persistent periapical periodontitis [3]. Recent metagenomic sequencing of intraradicular infectious microorganisms also showed that *E. faecalis*, *Streptococcus gordonii*, *Actinomyces naeslundii*, and *Lactobacillus acidophilus* are most frequently detected in secondarily infected root canals [4–6]. Therefore, *E. faecalis* is considered as a major pathogen, causing various community infections and endodontic failures. The important role of *E. faecalis* in various infection diseases is attributed to its strong resistance to many antibiotics [7]. As well as resistance to the antimicrobial drugs used for treatment, *E. faecalis* has evolved tolerance for various environments, especially its adaptation to withstand various pHs [8, 9]. Macrophages play a crucial role in bacterial infections, but *E. faecalis* survives well after its internalization in macrophages [10]. Bacteria are internalized by phagocytes and form early phagosomes, and phagosomal acidification creates a high-stress environment and impedes microbial growth [11]. The pH value of phagosome is about 5.5-6.0 and the luminal pH can reach as low as 4.5 after phagosome fused with lysosomes and generated phagolysosome [11]. However, *E. faecalis* is resistant to low pH in vivo and is capable of surviving within macrophages for long periods [12]. Thus, *E. faecalis* contributes to chronic local or systemic infection diseases. Therefore, elucidating the survival mechanism of *E. faecalis* under low-pH conditions is the key to combating the infectious diseases caused by *E. faecalis.*

As lactic acid bacteria (LAB), the enterococci also have the characteristics common to all LAB. They are Gram-positive, non-spore-forming, microaerophilic or anaerobic bacteria that produce lactic acid as the major end product of sugar fermentation [13]. Therefore, the physiology of enterococci under stress is similar to that of other LAB. The genes encoding ClpP and Clp ATPase are by far the best studied streptococcal stress genes in terms of their virulence potential. In *S. mutans*, which directly links low pH with dental caries, Clp is considered to play an important role in the acid stress response [14]. According to previous studies [15, 16], *E. faecalis* has strong resistance to high-pH stress according to ATPase activity. It carries genes encoding ClpP, ClpB, ClpC, ClpE, and ClpX, but the involvement of ClpP and Clp ATPase in resistance to acid stress has not been studied extensively in *E. faecalis*.

To identify genes potentially involved in essential bacterial survival or growth under various conditions, transposon insertion sequencing (TIS) has been optimized for many different bacterial species [17].

In this study, we used TIS to analyze *E. faecalis* strain OG1RF to identify genes related to its acid tolerance mechanism. Collectively, our findings show that metabolic adaptations are essential for the acid tolerance of *E. faecalis*.

## Methods

### Bacterial culture

*Enterococcus faecalis* strain OG1RF was used throughout this study. It was cultured in M9 broth (SIGMA, St. Louis, MO, USA) at pH 5 or pH 7 (M9 represent pH7-M9 if not specifically labeled in this article) at 37 °C in an aerobic atmosphere. The pH of the M9 broth was adjusted with HCl or NaOH. Samples (14 mL) of M9 broth (pH 7 and pH 5) were inoculated with 10^8^ colony-forming units (CFUs)/mL *E. faecalis* OG1RF, which were cultured at 37 °C until stationary phase.

### RNA-seq

After 12 h of cultivation, the cultures were centrifuged at room temperature at 2000 rpm for 15 s, and the pellets were flash frozen in liquid N_2_ before RNA extraction, which was performed as described previously [18]. The ScriptSeq Complete Kit (Bacteria) (Epicentre Biotechnologies, Madison, WI, USA) was used to remove ribosomal RNA (rRNA) and to construct a strand-specific library. Briefly, rRNA was removed from 2.5 µg of total RNA. To generate strand-specific RNA-seq data, approximately 100 ng of rRNA-depleted RNA was fragmented and reverse transcribed with random primers containing a 5′ tagging sequence, followed by 3′ end tagging with a terminal-tagging oligonucleotide to yield dual-tagged, single-stranded cDNA. After magnetic-bead-based purification, the dual-tagged cDNA was amplified with PCR (15 cycles) using ScriptSeq Index PCR Primers (Epicentre Biotechnologies). The amplified RNA-seq libraries were purified with the AMPure XP system (Beckman Coulter, San Jose, CA, USA) and sequenced as 100-bp paired-end reads on the Illumina HiSeq 2500 platform (University of Edinburgh, UK). The data were analyzed was with Rockhopper [19], using the default settings for strand-specific analyses.

### TIS

A transposon insertion mutant library of *E. faecalis* OG1RF was constructed with a mariner transposon, as described by Wei et al. [20]. Briefly, the transposon was delivered by pZXL5 plasmid carrying a chloramphenicol (Cm)-resistance gene. The pZXL5 plasmid was loaded into *E. faecalis* OG1RF with electroporation, and the transformants were cultivated BHI agar plates (containing both gentamicin and chloramphenicol) at 28 °C overnight. Then the bacteria carrying pZXL5 were incubated in pH5-M9 or M9 at 42 °C for 24 h. The mutant library was amplified in BHI medium with gentamicin at 37 °C.

To identify the genes essential for the survival of *E. faecalis* in pH5-M9, high-throughput sequencing and analysis was performed according to previous studies [21, 22]. The bacteria from the input and output libraries were collected, and genomic DNA were extracted with the Bacterial Genomic DNA Extraction Kit (Takara). The DNA was fragmented by sonication, end-repaired with A tailing and addition of adaptors and P5 and P7 sequences by two cycles of PCR. Three replicates of the input and output libraries were sequenced with high-throughput sequencing on the Illumina HiSeq 2500 platform. The sequence reads were mapped to the genome, and the mapped read-counts were then tallied for the analysis of the essentiality of the genes in the *E. faecalis* OG1RF genome. The read counts for each locus were normalized among the three libraries according to the sequencing depth. The fold change of each locus was generated by comparing the output read counts with the input read counts. The essential loci were determined with the HMM module of EL-ARTIST [23].

### Data analysis

The raw Illumina MiSeq sequencing data were split, based on their barcodes, using the Galaxy platform, and 16-nt fragments of each read that corresponded to an OG1RF sequence were mapped to the OG1RF genome with Bowtie 2 [24]. Reads that mapped to the final 10% of genes were discarded because such insertions may not inactivate the gene function. Feature Counts were used to determine the read counts of the transcripts. The read counts per gene were then normalized to the total number of reads that mapped to the genome in each replicate, by calculating the normalized reads per kilobase per million input reads (RPKM) with the following formula: RPKM = (number of reads mapped to a gene × 10^6^)/(total mapped input reads in the sample × gene length in kbp) [25]. Statistical analysis of the differences in the RPKM values under each experimental condition was performed with Cyber-T [26]. A difference analysis of the groups was performed with DEseq2. Genes were deemed to contribute significantly to bacterial growth when the Benjamini–Hochberg-corrected *P* value was < 0.05 and the difference in abundance of the transposon mutant during growth in M9 and in serum was > 2 [25]. Gene ontology (GO) and Kyoto Encyclopedia of Genes and Genomes (KEGG) [27–29] enrichment analyses were performed with the R package. Other statistics were calculated and plots drawn with Perl and Python scripts.

The M9 group was used as the control for both RNA-seq and TIS. For RNA-seq, differential genes were filtered with |log_2_FC|, i.e., a gene change was deemed reliable at |log_2_FC| > 1 and adjusted *P* ≤ 0.05. For TIS, expression changes were deemed reliable at adjusted *P* ≤ 0.05 only.

## Results

### RNA-seq and TIS data analyses

**Fig. 1:**
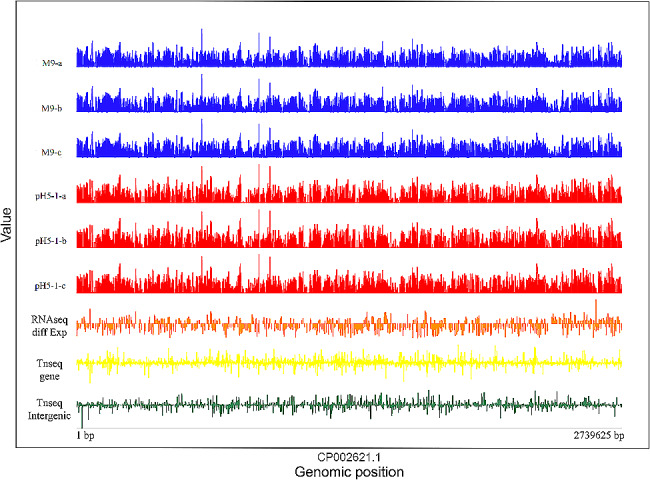
Transcriptomic analysis of E. faecalis. The y-axis of each track indicates read coverage and is represented on a log scale, ranging from 0 to 10,000. The x-axis represents the genomic location. The blue (M9-1) and red (pH5-1) tracks correspond to sequencing reads for E. faecalis cultured at different pHs, and a, b and c represent three replicates. The orange (RNAseq diff Exp) track corresponds to genes differentially expressed between two groups, and the heights of the bars indicate expression levels. The yellow (Tn-seq) and dark green (Tn-seq intergenic) tracks represent genes and intergenic regions detected with Tn-seq, respectively. The RNA-seq experiments were performed with three biological replicates

To identify the genes of *E. faecalis* required for growth in acid environments, TIS of cultures of *E. faecalis* grown in M9 broth at pH 7 and pH 5 was performed. To ensure that *E. faecalis* was able to survive and multiply when incubated at pH 5, the transcriptional profile of the strain was determined during exponential growth phase in M9 broth and M9 broth at pH 5. The read count values of two groups and three replicates are shown in Fig. 1. The |log_2_FC| of RNA-seq differential expression, TIS gene and TIS intergenic were presented.

**Fig. 2:**
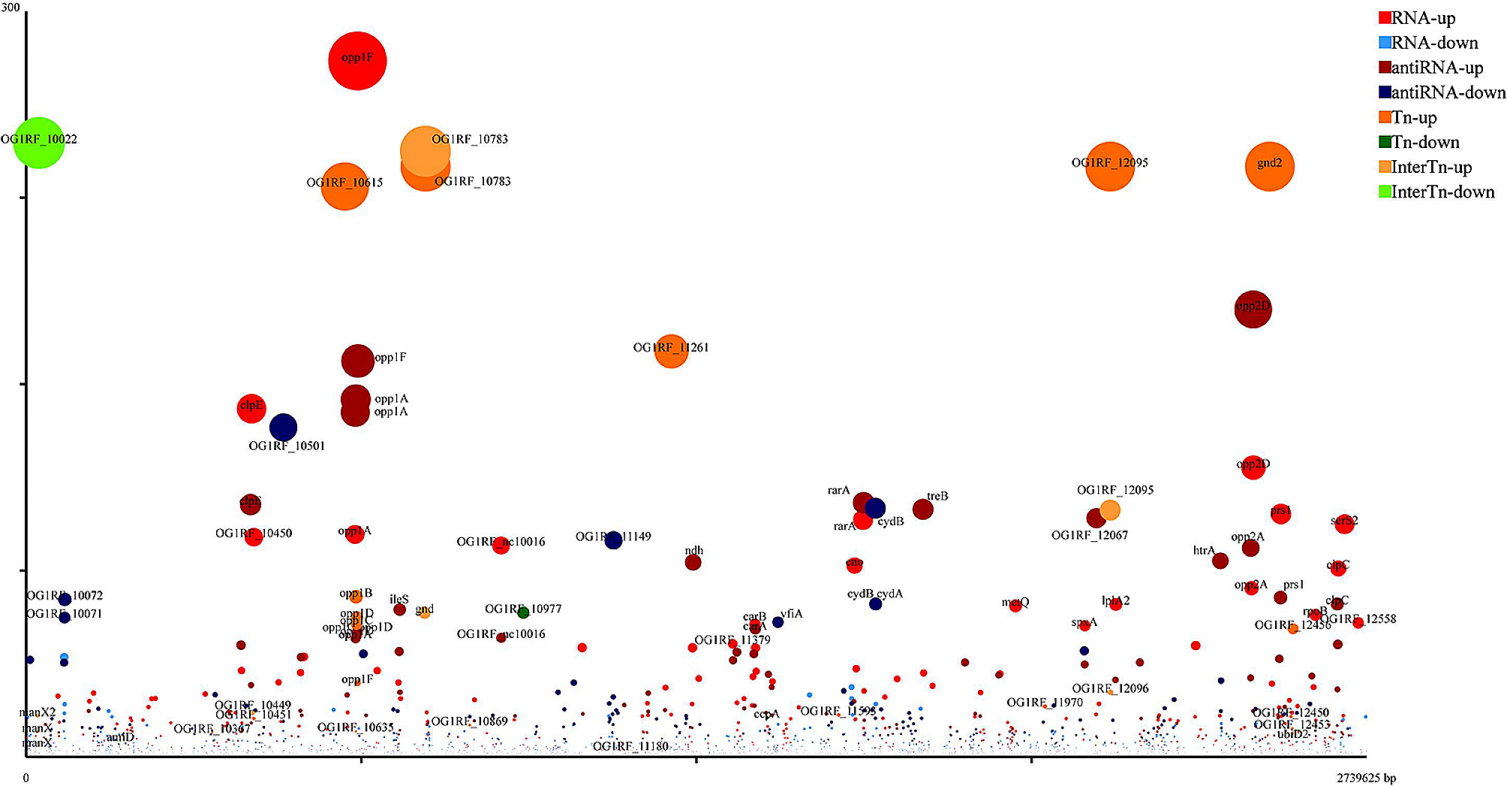
Crosslink analysis to determine the genes required for the acid tolerance of E. faecalis. Bubbles represent genes, and their sizes indicate fold-changes in their expression compared with their expression in E. faecalis in M9 broth. The x-axis shows the genomic locations of the genes. The y-axis showed the − log10(q), representing the degree of variance. Significantly differentially expressed (q < 0.05) genes are labeled with their names in different colors, indicating whether they were upregulated or downregulated on RNA-seq or Tn-seq

The upregulated and downregulated genes detected with RNA-seq and TIS are shown in Fig. 2. A total of 364 genes were upregulated and 615 were downregulated.

### Upregulated genes detected with RNA-seq

**Fig. 3:**
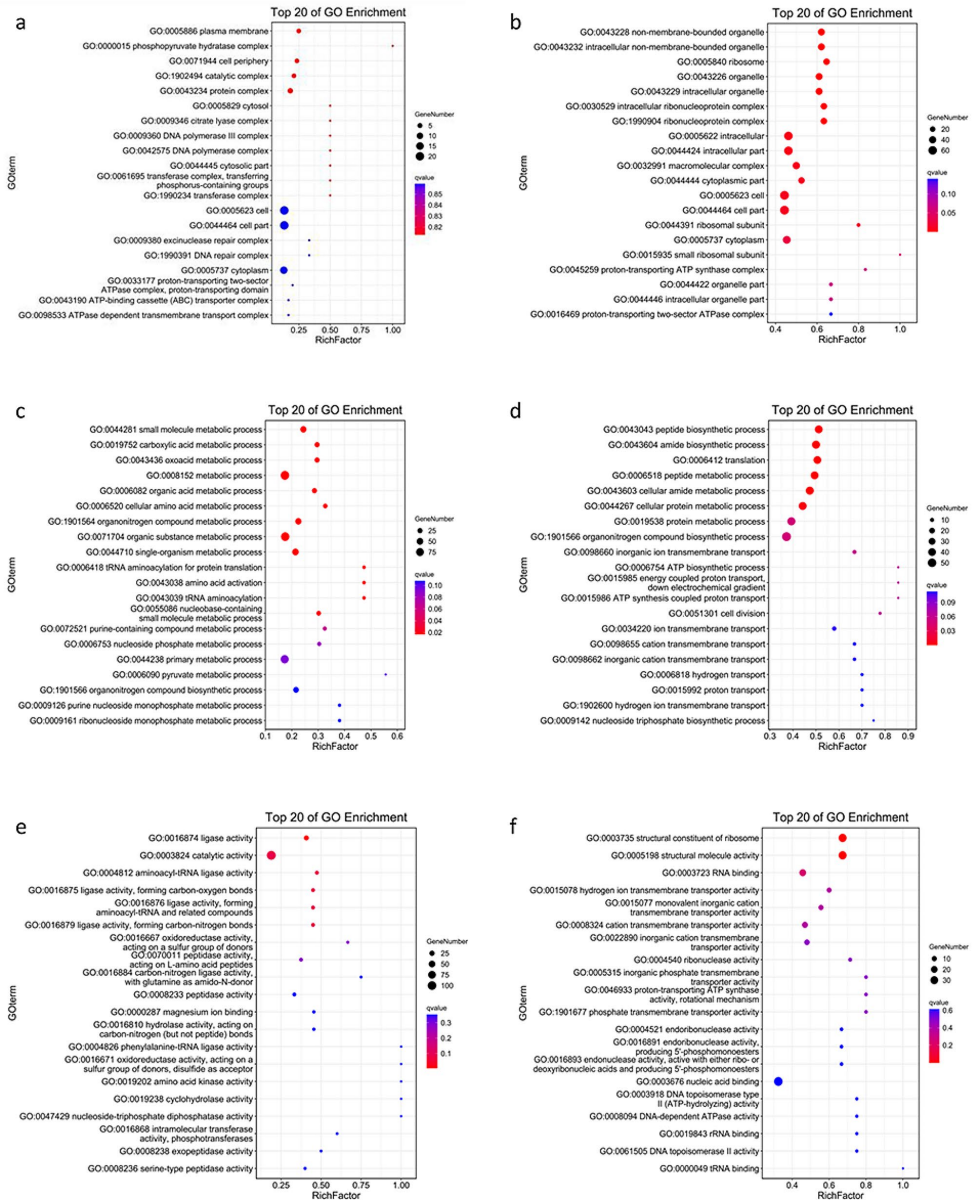
Upregulated and downregulated genes detected with RNA-seq, according to GO enrichment. (**a**) Upregulated genes involved in “cellular component” category. (**b**) Downregulated genes involved in “cellular component”. (**c**) Upregulated genes involved in “biological progress”. (**d**) Downregulated genes involved in “biological progress”. (**e**) Upregulated genes involved in “molecular function”. (**f**) Downregulated genes involved in “molecular function”

Multiple aspects of function were identified among the 364 upregulated genes detected with RNA-seq (Fig. 3). The results of a GO analysis indicated that the genes that conferred acid tolerance on *E. faecalis* included those in the categories “biological process”, “molecular function”, and “cellular component”.

The genes categorized in “biological process” included some involved in carboxylic acid metabolic processes and oxoacid metabolic processes (*OG1RF_11855*, *argS*, *carA*, *folA*, *folE*, *gdhA*, etc.). Other genes involved in cellular amino acid metabolic processes and organonitrogen compound metabolic processes (*serS2*, *srpG2*, *thiN*, *thrB*, *thrS*, *valS*, etc.) also accounted for a large proportion of differentially expressed genes. In the “molecular function” category, a number of genes associated with enzyme activity were upregulated, mainly ligases, catalytic enzymes, and especially those forming carbon–nitrogen bonds, etc., indicating an increase in the metabolic rate when *E. faecalis* encountered an acid environment. Among the genes mentioned above, ligase-activity-related genes were significantly more enriched than other differentially expressed genes. However, the enrichment of plasma-membrane-associated genes surpassed that of others genes in the “cellular component” category. Other genes linked to the plasma membrane and DNA polymerase were also enriched, indicating a potential association between acid tolerance and both the plasma membrane and gene repair.

**Fig. 4:**
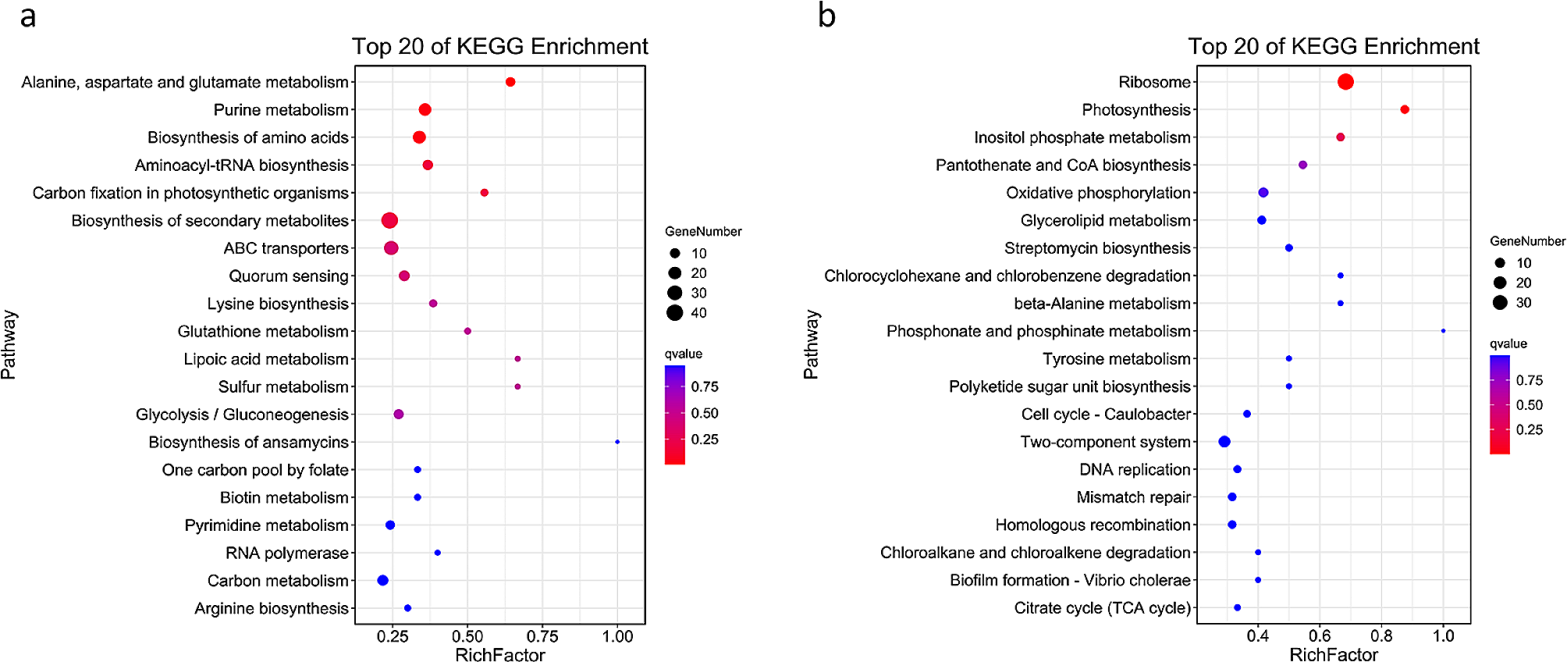
Upregulated and downregulated pathways detected with RNA-seq according to their KEGG enrichment. (**a**) Upregulated pathways enriched in KEGG analysis. (**b**) Downregulated pathways enriched in KEGG analysis

In a KEGG analysis (Fig. 4), the pathways associated with alanine, aspartate, and glutamate metabolism, purine metabolism, and the biosynthesis of amino acids showed significant enrichment (q < 0.05), and pathways involving aminoacyl-tRNA biosynthesis, carbon fixation in photosynthetic organisms, the biosynthesis of secondary metabolites, ABC transporters, and quorum sensing were also enriched (*p* < 0.05).

### Downregulated genes detected with RNA-seq

In the GO and KEGG analyses, the downregulated genes were categorized in the same three categories as the upregulated genes (Figs. 3 and 4).

Genes involved in the processes of “cellular protein metabolism”, “cellular amide metabolism”, and “peptide biosynthesis and metabolism” were predominantly enriched. This suggests that cellular amino acids were taken up and metabolized to neutralize the excess hydrions in the acid environment. In terms of “molecular function”, proteins involved in the transmembrane transport of hydrions and other cations were also categorized, suggesting that the cation transportation capacity was downregulated, probably to balance the potential difference across the membrane. In terms of “cellular components”, the expression of genes involved in non-membrane-bounded organelles were mainly downregulated.

A KEGG analysis showed that the downregulated genes were involved in the pathways: “ribosome”, “inositol phosphate metabolism”, “pantothenate and CoA biosynthesis”, and “oxidative phosphorylation”.

### Differentially expressed genes detected with both RNA-seq and TIS

**Fig. 5:**
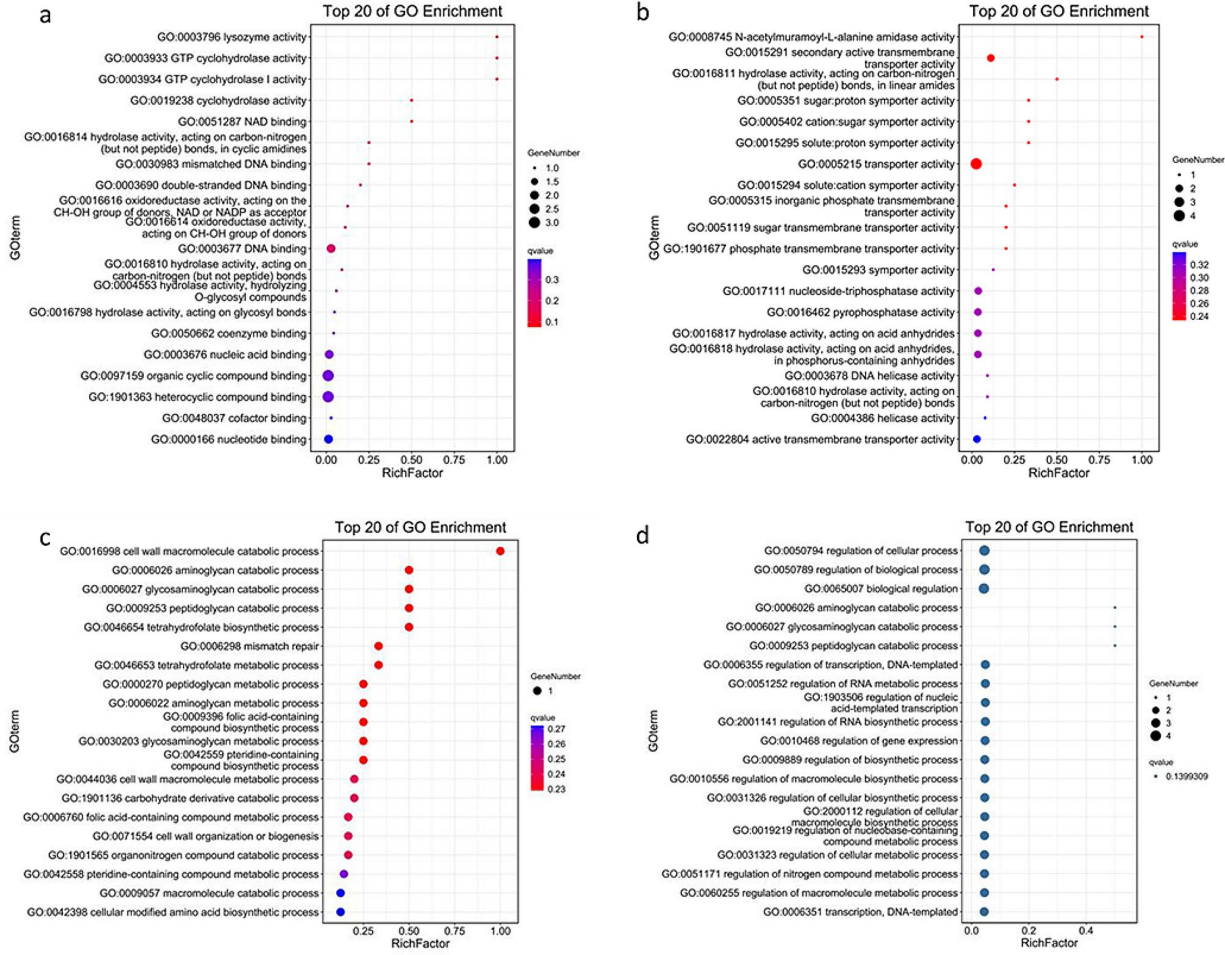
Upregulated and downregulated genes detected with both RNA-seq and Tn-seq according to GO enrichment. (**a**) Upregulated genes involved in “molecular function” detected with both RNA-seq and Tn-seq. (**b**) Downregulated genes involved in “molecular function” detected with both RNA-seq and Tn-seq. (**c**) Upregulated genes involved in “biological progress” detected with both RNA-seq and Tn-seq. (**d**) Downregulated genes involved in “biological progress” detected with both RNA-seq and Tn-seq

**Fig. 6:**
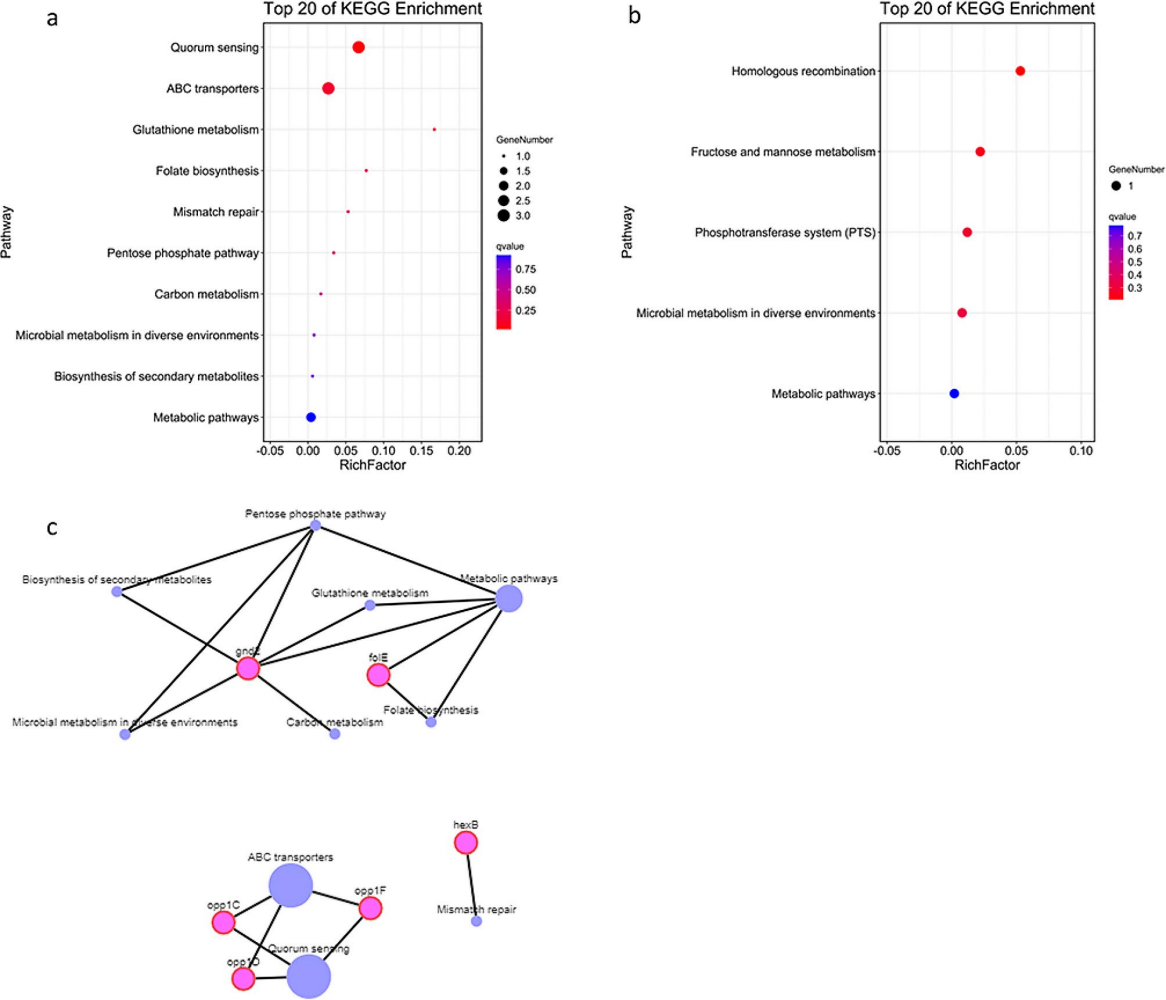
Upregulated and downregulated pathways detected with both RNA-seq and Tn-seq according to KEGG enrichment. (**a**) Upregulated pathways detected with both RNA-seq and Tn-seq and analyzed with KEGG. (**b**) Downregulated pathways detected with both RNA-seq and Tn-seq and analyzed with KEGG. (**c**) Map of genes and pathways upregulated in the KEGG analysis. Purple indicates pathways and pink indicates genes; larger circles indicate lower p values

When the differentially expressed genes detected with RNA-seq or TIS were compared, those detected with both analyses were identified and are shown in Figs. 5 and 6. Seventeen differentially expressed genes were analyzed with GO and KEGG. The genes were divided into three categories according to function. The GO enrichment identified *opp1C, OG1RF_11718*, *folE*, *OG1RF_12404*, *hexB*, etc. in the “cellular component” and “molecular function” categories, and the KEGG analysis identified *opp1C*, *opp1D*, and *opp1F* as associated with the ABC transporter pathway, consistent with the GO analysis.

All 17 genes, (*opp1C*, *opp1D*, *opp1F*, *copY*, *gldA2*, *ubiD*, *ubiD2*, *folE*, *lyzl6*, *hexB*, *gnd2*, *OG1RF_11464*, *OG1RF_11718*, *OG1RF_12453*, *OG1RF_10680*, *OG1RF_10635*, and *OG1RF_12404*) are mainly involved in functions that allow survival under stress condition, as further analyzed in discussion part.

## Discussion

To understand acid resistance and its mechanism in microorganisms requires the investigation of how they interact with their environments and the adaptive modifications that allow them to do so. Species such as *Escherichia coli* and *Salmonella enterica* are highly resistant to acid conditions and can survive at very low pHs, including in the mammalian stomach [30]. The acid-resistance mechanisms of microorganisms allow them to survive extreme acid pressure. One of these mechanisms involves the manipulation of the H^+^ transport system to maintain a lower intracellular concentration of protons. Other bacteria resist acid stress by synthesizing alkaline products that neutralize environmental acids [31]. Their activities of sensing, response, and adaptation to acid stress are involved in the acid tolerance response.

In this study, acid stress was applied by the direct addition of HCl to the culture medium to reduce the pH, after which we investigated the genes help *E. faecalis* to survive in acid conditions. To identify such genes, we used both RNA-seq and TIS. Among the genes differentially expressed under acid conditions, *opp1C* was enriched in the “cellular component” category. Opp1 acts as a metal transporter in *Staphylococcus aureus*, transporting cobalt and nickel together in the ABC transporter system [32]. The transportation of metal cations may contribute to the balance of the transmembrane potential, maintaining a rising concentration of potential to prevent excess acidification.

The differentially expressed genes in the “molecular function” category included *OG1RF_11718*, *folE*, *OG1RF_12404*, and *hexB*. The *folE* gene encodes zinc-dependent GTP cyclohydrolase IA in *Bacillus subtilis*, which participates in folate biosynthesis from the metabolism of GTP [33]. This suggests that an acid environment encourages microbes to produce folate to deal with the consequent impairment of genes. The repair function of the mismatch elimination protein HexB has been studied in *S. pneumoniae* [34], and may co-operate with other pathways to guarantee DNA stability in *E. faecalis*. According to our GO analysis, *OG1RF_11718* is related to lysozyme activity, and *OG1RF_12404* may be associated with nicotinamide adenine dinucleotide (NAD) binding and oxidoreductase activity, acting on the CH-OH group of donors. A previous study showed that lysozyme bound RisV of *E. faecalis*, triggering a signal transduction cascade that reduced the activation of extracytoplasmic function (ECF) σ factor σ^v^, thus regulating the lysozyme resistance of the bacterium [35]. The upregulation of lysozyme may be useful in consuming the excess cations present in an acid environment. NAD and NADP are closely associated with nitroreductase, a member of the oxidoreductase family, which reduces nitro compounds, ultimately to amino compounds [36]. Therefore, OG1RF_12404 probably contributes to amino acid metabolism and the further neutralization of acid. Also, the down-regulation of *clpX* showed its consistency with previous study [14]. *clpX* encoded a protein that interact with clpP to function. The deletion of *clpX* caused an enhanced resistance to acid killing in *S.mutans*, which may be attributed to the dysfunction of clpPX and its downstream regulatory protein, and its slower metabolism affected by clpX [14]. And the down-regulation of *clpX* in OG1RF functioned probably the same.

The biological processes affected by acid stress included “cell-wall macromolecule catabolic processes”, “aminoglycan catabolic processes”, and “mismatch repair”. When the genes shown to be upregulated with both RNA-seq and Tn-seq were considered, we concluded that macromolecule catabolism may involve the metal cation transporter Opp1, the ABC transporters, and other unknown proteins that function in defensive mechanisms. OG1RF_12404 is probably involved in aminoglycan catabolism, whereas FolE and HexB are involved in mismatch repair. In our KEGG analysis, *opp1C*, *opp1D*, and *opp1F* were linked with ABC transporters, consistent with the GO analysis. The glutathione-metabolism-related gene *gnd2* encodes a 6-phosphogluconate dehydrogenase, which catalyzes the conversion of glucose 6-phosphate to ribulose 5-phosphate [37]. Therefore, Gnd2 is involved in the generation of NADPH and other metabolisms of reduction progress.

The significantly differentially expressed genes detected with both RNA-seq and Tn-seq are discussed above. However, other genes also play a part in acid tolerance. CopY is a copper repressor in the copper export system (cop operons), suggesting a function similar to that of Opp1C [38]. Research into *gldA* in *E. coli* may clarify its role in *E. faecalis* [39]. The *gldA* gene encodes glycerol dehydrogenase, which converts glycerol to dihydroxyacetone (DHA), and regulates the intracellular levels of DHA, further affecting metabolism. The *ubiD* and *ubiD2* genes encode UbiD enzymes, which activate (hetero)aromatic C–H decarboxylation under ambient conditions, providing a route to the corresponding acids and derivative compounds [40].

In conclusion, we used RNA-seq and Tn-seq to identify the genes in *E. faecalis* required for its acid tolerance, highlighted genes like *opp1C*, *copY*, *gnd2*, *gldA*, *ubiD*, *ubiD2*, *folE* and *hexB*, implicating a possible network of co-operating factors described above in its resistance to acid environments. Based on these results and previous reports [32, 38], we speculated that the high H + concentration activates the expression of opp1C and copY to maintain the balance of the transmembrane potential by importing metal irons and regulating hydrion concentration. And *gnd2* regulate the generation of NADPH and *OG1RF_12404* probably regulates aminoglycan metabolism via NAD binding, together ensuring the adequate neutralization of excess acids. In addition, *folE* up-regulates folate biosynthesis and *hexB* helps with mismatch elimination protein, enhancing the genetic stability of *E. faecalis* in acidic environments. Further studies are needed to prove the specific role of the identified genes in surviving in acid stress.

## Data Availability

All raw sequences were deposited in the NCBI Sequence Read Archive (BioProject: PRJNA960628).
